# Dynamic Collision Behavior Between Osteoblasts and Tumor Cells Regulates the Disordered Arrangement of Collagen Fiber/Apatite Crystals in Metastasized Bone

**DOI:** 10.3390/ijms19113474

**Published:** 2018-11-05

**Authors:** Aira Matsugaki, Tatsuki Harada, Yumi Kimura, Aiko Sekita, Takayoshi Nakano

**Affiliations:** Division of Materials and Manufacturing Science, Graduate School of Engineering, Osaka University, Suita 5650871, Japan; matsugaki@mat.eng.osaka-u.ac.jp (A.M.); tatsuki.harada@mat.eng.osaka-u.ac.jp (T.H.); yumi.kimura@mat.eng.osaka-u.ac.jp (Y.K.); aiko.sekita@mat.eng.osaka-u.ac.jp (A.S.)

**Keywords:** bone metastasis, bone microstructure, collagen/apatite, cell arrangement

## Abstract

Bone metastasis is one of the most intractable bone diseases; it is accompanied with a severe mechanical dysfunction of bone tissue. We recently discovered that the disorganized collagen/apatite microstructure in cancer-bearing bone is a dominant determinant of the disruption of bone mechanical function; disordered osteoblast arrangement was found to be one of the principal determinants of the deteriorated collagen/apatite microstructure. However, the precise molecular mechanisms regulating the disordered osteoblast arrangement triggered by cancer invasion are not yet understood. Herein, we demonstrate a significant disorganization of bone tissue anisotropy in metastasized bone in our novel ex vivo metastasis model. Further, we propose a novel mechanism underlying the disorganization of a metastasized bone matrix: A dynamic collision behavior between tumor cells and osteoblasts disturbs the osteoblast arrangement along the collagen substrate.

## 1. Introduction

Despite significant developments in bone metastasis research over the past decades, strategies for the recovery of fully-functionalized bone tissue from diseased bone, which can realize the required mechanical properties, has remained elusive. Recently, we removed the veil of the deepest mystery of bone metastasis: “Why cancer weakens the targeted bone separately from the alteration in bone mass”. The bone metastasis procedure is strictly regulated by the complicated dialogues between cancer cells and bone metabolism [1,2,3]. The mechanical dysfunction accompanied with the increased risk of pathological fracture occurs both in osteoblastic and osteolytic metastasis, indicating that the alteration in bone mass cannot explain the mechanical disorders induced by cancer involvement. Intact bone tissue exhibits a characteristic anisotropic microstructure based on collagen fiber alignment and the related *c*-axis orientation of apatite crystals [4,5], which is derived from the intercellular molecular events mediating the cellular arrangement [6]. We recently discovered that the disorganized, less-aligned collagen/apatite microstructure in cancer-bearing bone is a dominant determiner in the disruption of bone mechanical function [7,8]. However, the biological scenarios that stimulate the disorganization of metastasized bone microstructures are not fully understood.

To date, studies involving in vitro engineering approaches have been carried out to elucidate bone metastasis, and currently, various 3D approaches to model bone metastasis have been developed. These biosystems have aided the understanding of the spatial interactions between multiple cell types and the bone microenvironment, which involve the relationship between angiogenesis and metastasis [9], and the chemosensitivity of cells to therapeutic agents [10,11]. Although these approaches are intriguing, knowledge regarding the structural interactions between cancer metastasis and bone environment are lacking. Here, we have developed a novel ex vivo bone metastasis model that allows the analysis of the bone anisotropic microstructure related to cancer metastasis, and have demonstrated its pioneering role in the elucidation of molecular cues involved in the disarrangement of the collagen/apatite in metastasized bone. Further time-lapse imaging of an in vitro anisotropic coculture model unveiled how the adhesive interactions with cancer cells disorganize the osteoblast arrangement, and further, the construction of deteriorated bone tissue.

## 2. Results

### 2.1. Establishment of an Ex Vivo Bone Metastasis Model

**Figure 1:**
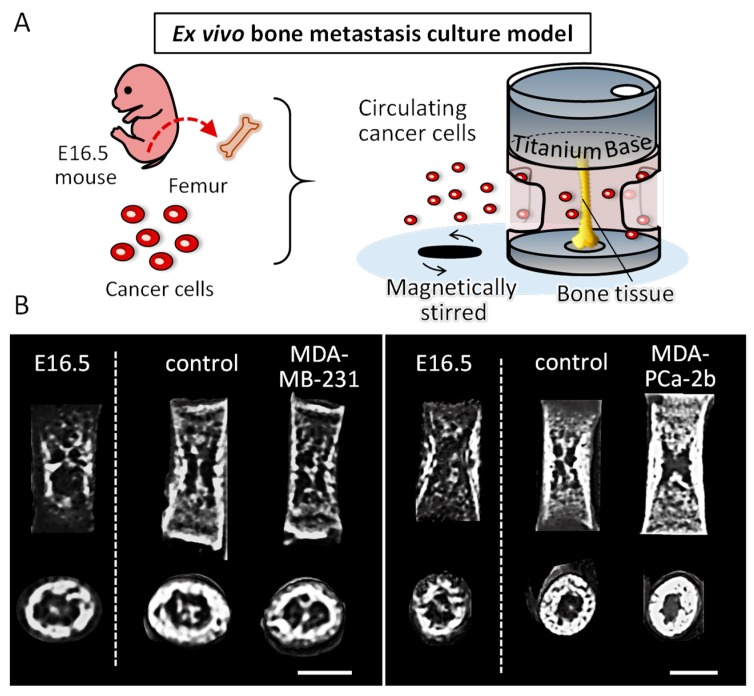

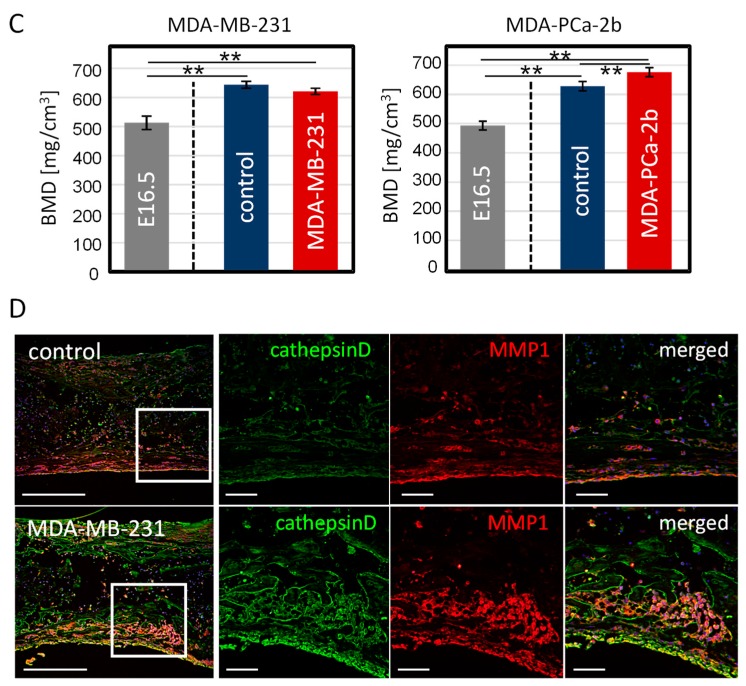
(**A**) Schematic illustration of an ex vivo bone metastasis culture model. E16.5 mouse femurs were cultured inside the static longitudinal loading device with circulating cancer cells. (**B**) Micro-CT images of the femurs before cultivation (E16.5) and after cultivation with or without tumor cells. Scale bars: 0.5 mm. (**C**) Bone mineral density (BMD) analysis of the femurs before cultivation (E16.5) and after cultivation (separated by dot lines) with or without tumor cells. (**D**) Immunohistochemical analysis of the cultured femurs with and without breast cancer MDA-MB-231 cells. Magnified images of the enclosed regions are shown on the right side. Green; cathepsin D, Red; MMP1. Scale bars: 100 µm. ** *p* < 0.01.

A novel ex vivo bone metastasis model was successfully constructed by the controlled culturing of embryonic bone tissue inside a static uniaxial loading device (Figure 1A). E16.5 mice femurs showed an increased bone mineral density (BMD) after being cultured for seven days (E16.5 vs. control) (Figure 1B,C). In addition, micro-CT images showed an increased bone length in cultured femurs as compared to the femurs before cultivation, indicating that the cultivation under static loading condition promoted the longitudinal growth of embryonic femurs during cultivation. Bone tissue culturing with circulating tumor cells resulted in distinct features corresponding to the type of cancer cells. Coculture with osteolytic breast cancer cells demonstrated a tendency of decreased BMD, whereas coculture with osteoblastic prostate cancer cells displayed an increased BMD. Micro-CT images indicate that the longitudinal growth of femurs is not significantly affected by the coculture with cancer cells, on the other hand, the images of the horizontal section of femurs indicate the increased bone growth in the endosteal side in prostate cancer MDA-PCa-2b invaded bone. Immunohistochemical analysis revealed that the breast cancer cell markers cathepsin D and MMP1 (matrix metalloproteinase 1) were highly expressed in the cocultured bone (Figure 1D), indicating that our ex vivo metastasis model mimics the biological events triggered by cancer invasion. The tumor cells likely invaded into the cultured bone tissue during the cultivation period, as the colonization of tumor cells was not detected on the bone surface after seven days of cultivation.

### 2.2. Disorganized Bone Microstructure of Tumor-Invaded Bone in Organ Culture

**Figure 2:**
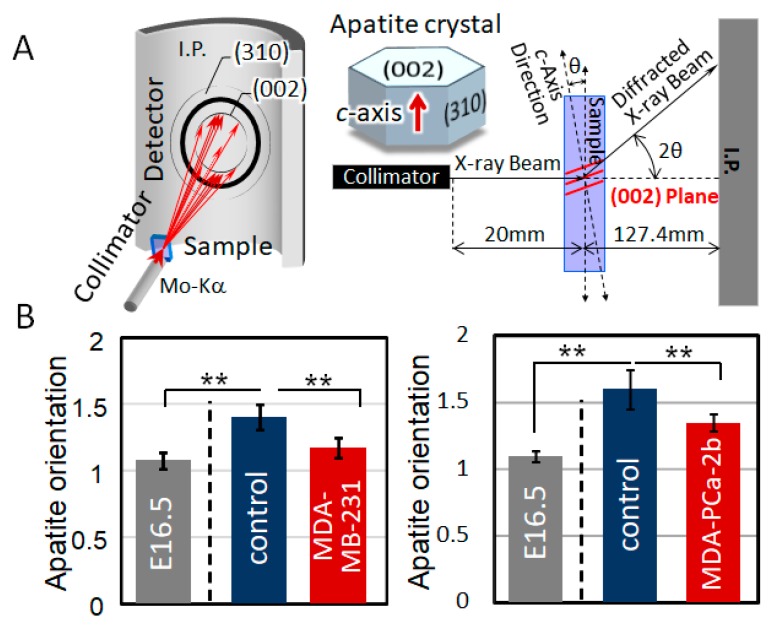
(**A**) Schematic illustration of the analysis of apatite orientation using transmission microbeam XRD (X-ray diffraction) method. Preferential orientation of the *c*-axis of apatite crystals was analyzed with the integrated intensity ratio of (002)/(310). (**B**) Quantitative analysis of apatite orientation along the longitudinal direction of the bone before cultivation (E16.5) and after cultivation (separated by dot lines) with or without tumor cells. ** *p* < 0.01.

**Figure 3:**
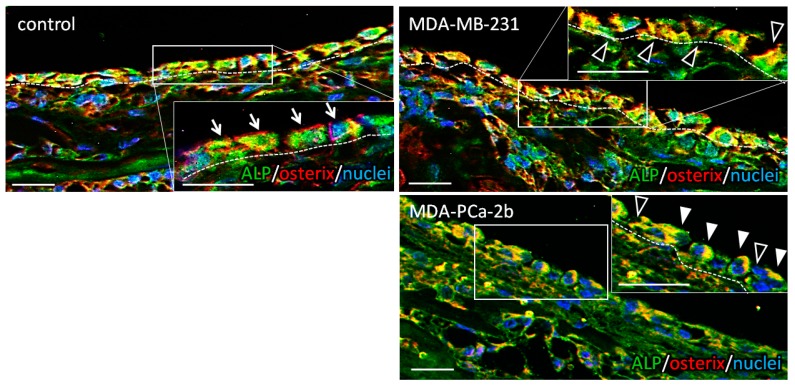
Immunohistochemical images of the bone surface cultured without tumor cells (control), with breast cancer MDA-MB-231 cells, and prostate cancer MDA-PCa-2b cells. Insets show magnified images. Green; ALP (alkaline phosphatase), Red; osterix, Blue; nuclei. Arrows indicate the ordered osteoblasts in control. Black arrowheads show disrupted intercellular connection; white arrowheads indicate the abnormal morphology of osteoblasts. Dot lines indicate the bone surface. Scale bars: 20 µm.

The µXRD analysis revealed that the bone tissue cocultured with tumor cells showed a disorganized microstructure with less-aligned apatite crystals in both osteoblastic metastasis and osteolytic metastasis (Figure 2). Intact femurs showed a significantly higher degree of apatite orientation compared to E16.5 femurs (before cultivation), which indicates that the highly organized collagen/apatite matrix was constructed in response to the external mechanical stimuli. The immunohistochemical analysis of cultured bone tissue showed that the osteoblasts were arranged in line on the surface of the control bone, whereas they showed a disorganized cell alignment in cocultivation with tumor cells (Figure 3). Quantification of the tumor cell-induced osteoblast disarrangement will be conducted in our next study, which will definitely help provide a better understanding of the importance of osteoblast alignment in the regulation of anisotropic bone tissue geometry.

### 2.3. Dynamic Interaction with Tumor Cells Induces the Impaired Alignment of Osteoblasts

**Figure 4:**
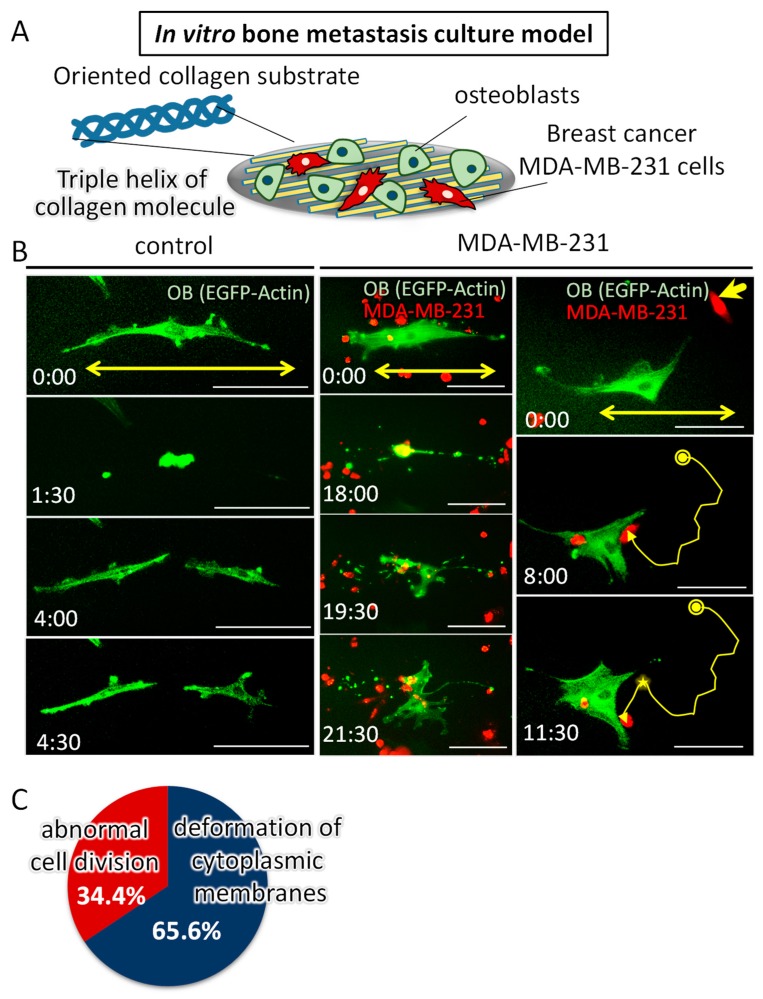
(**A**) Schematic illustration of the in vitro bone metastasis culture model. Osteoblasts and breast cancer MDA-MB-231 cells were cocultured on an artificially controlled oriented collagen substrate. (**B**) Time-lapse imaging of the monocultured osteoblasts (control), and cocultured osteoblasts and MDA-MB-231 cells. Green; EGFP-Actin (osteoblasts), Red; CellTracker (MDA-MB-231 cells). The yellow double-headed arrows indicate the substrate collagen orientation. The yellow arrows with yellow circles indicate the locomotion traces of MDA-MB-231 cells. The yellow circles indicate the initial position of the MDA-MB-231 cell represented by the bold yellow arrow. The star mark indicates the intercellular collision position. Scale bars: 100 µm. (**C**) Quantitative analysis of the frequency of the observed two types of dynamic collision behavior that lead to the disrupted alignment of osteoblasts.

Time-lapse imaging revealed that the dynamic interaction with tumor cells influenced the osteoblast morphology and alignment on oriented collagen substrates (Figure 4). The osteoblasts directly “touched” by MDA-MB-231 cells failed to align along the collagen substrate, whereas the intact osteoblasts showed a preferential alignment along collagen substrate. The disruption dynamics were found to be classified into mainly two types of interactions: The disorganization of the cell division axis along the collagen orientation triggered by a tumor cell attack, and the deformation of cytoplasmic membranes triggered by the dynamic collision with tumor cells. The frequency of these two types of dynamics was quantitatively analyzed; the osteoblast arrangement was disrupted via the abnormal cell division process (34.4%) and the deformation process of cytoplasmic membranes (65.6%).

## 3. Discussion

**Figure 5:**
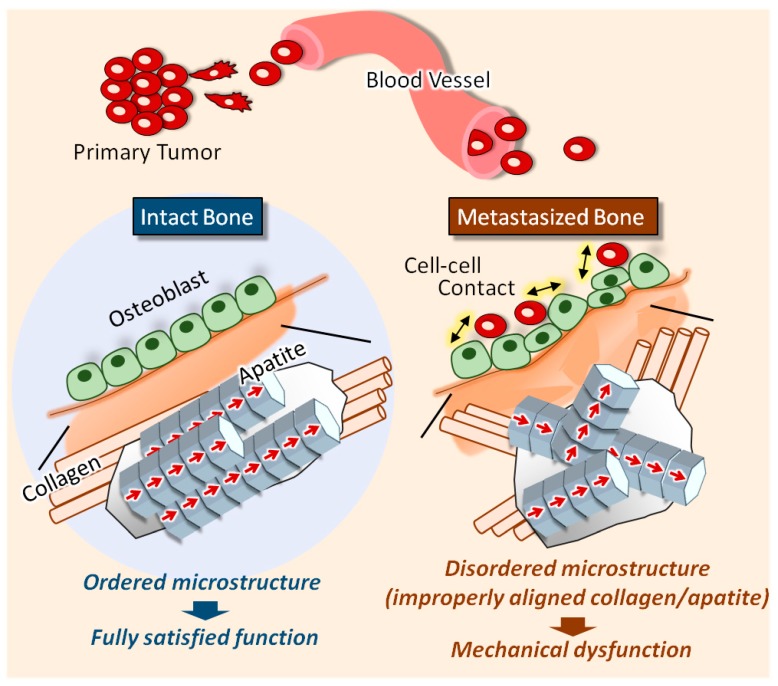
Schematic illustration of the deterioration of the collagen/apatite orientation in metastasized bone. The intact bone tissue shows the preferred orientation of collagen fibers and the *c*-axis orientation of apatite crystals along the longitudinal direction of long bones. Metastasized bone shows a disrupted collagen/apatite orientation, which is correlated to the disarrangement of osteoblasts on the bone surface.

The traditional understanding of bone metastasis has been limited to the pathological changes in bone accompanied with the alteration in bone mass. We recently pioneered a new avenue for bone metastasis treatment: Disordered microstructure in metastasized bone is the principal source of its mechanical dysfunction [7]. In the present study, we developed a novel organ culture model that mimics the physiological bone metastasis events, for the elucidation of the molecular mechanisms underlying the disruption of the ordered arrangement of the bone matrix. Furthermore, time-lapse imaging was used to visualize the intercellular dynamics mediating the impaired osteoblast arrangement between tumor cells and osteoblasts on controlled oriented collagen substrates (Figure 5).

To date, various kinds of biomimetic bone metastasis models involving spheroid cultures [12], microfluidic cell culture models [13], or 3D organ culture systems [14] have been constructed. These tumor engineering techniques have achieved certain results for understanding tumor growth characteristics and chemotherapy resistance. The limitation of these classical models is that they lack the native architecture of bone with regards to the surrounding mechanical environment. Our novel organ culture model overcomes these limitations of studying bone metastasis; it efficiently mimics the interaction of tumor cells and the ordered microstructure of bone tissue. The homing of cancer cells to bone is a multi-step process involving detachment from the primary tumor, invading inside the extracellular matrix, moving across the endothelium and taking up residence in the bone [15]. Our model mimics the later stage of these events which mimics the intercellular crosstalk between osteoblasts and invaded tumor cells; we focused on the interaction between tumor cells and osteoblasts in the bone microenvironment. Apatite orientation is closely related to the mechanical condition surrounding the bone, as indicated by our in vivo analysis [4]. Our ex vivo culturing system enables the static loading of femurs in a longitudinal direction. Embryonic femurs have no loading history, which enables the identification of their loading effects during the culture period. The femurs grew in a longitudinal direction in response to the static loading, with increased BMD (Figure 1B,C). These results indicate that the embryonic femurs showed healthy growth inside our culture device, in response to the external loading environment. The alteration of BMD in response to the coculturing with the different cancer cell types is considered to be derived from the regulation of osteoblast and osteoclast functions by soluble factors, and also their adhesive interactions with cancer cells [16]. In our model, the tumor cells adhere and invade the bone during cultivation for seven days. The observed effects on bone tissue are derived from both the soluble factors from the adhered tumor cells and also the direct interaction with invaded tumor cells. We have clarified that the cancer cell-derived soluble factors regulate the cell number and alkaline phosphatase expression in osteoblasts depending on the types of cancer cells in our previous report [17]. The osteolytic breast cancer cells (MDA-MB-231) and the osteoblastic prostate cancer cells (MDA-PCa-2b) were invaded into the intact femurs towards the bone surface using a three-dimensional cellular approach, resulting in pathological changes, as evidenced by the histological analysis (Figure 1D). Although the grafted tumor cells were not identified clearly on the bone surface, the strong expression of breast cancer markers cathepsin D and MMP1 near the bone surface indicate the successful tumorigenesis induced by cocultivation with tumor cells.

Apatite orientation is a next-generation index for bone diagnosis [18]; the traditional therapeutic index and bone mass cannot explain the bone functions. We have clarified that apatite orientation is the dominant determiner of bony mechanical function [19]. The ex vivo tumor-invaded bone showed a significantly disrupted alignment of apatite crystals (Figure 2), which is consistent with our previous in vivo reports [7,8,20]. Our ex vivo model can represent the alteration of bone apatite orientation in response to the external loading conditions [21]. Immunohistochemical analysis revealed that the ordered arrangement of intact osteoblasts with cuboidal morphology in line on the bone surface was significantly deteriorated by cancer cell invasion (Figure 3). On the MDA-PCa-2b-invaded bone surface, the osteoblasts show heterogeneous positioning, expressing an abnormal rounded or flattened shape with discrete intercellular connections. The abnormal disarrangement of osteoblasts with disrupted intercellular junctions was also observed on the MDA-MB-231-invaded bone surface. Osteoblast arrangement is one of the most significant contributors to the construction of an oriented bone matrix architecture [22]. We have clarified the quantitative coordination between osteoblasts and apatite orientation [6]. A clarification of what drives the deterioration of cell arrangement in metastasized bone can lead to the understanding of the molecular events controlling bone metastasis. Our in vitro-oriented bone metastasis model is a powerful tool for understanding the cellular response against the aligned microenvironment mimicking the anisotropic bone surface. Time-lapse imaging of the intercellular communication between osteoblasts and tumor cells on the oriented collagen substrates revealed that the dynamic cell-cell interaction plays key roles in regulating the osteoblast arrangement. We found mainly two types of cellular disorganization patterns: One is the abnormal cell division pattern and the other is the structural alteration of cytoplasmic membranes triggered by cell-cell interactions. Intact osteoblasts show a preferential alignment along the substrate collagen orientation via integrin receptors [23,24]. The cell division axis determines tissue polarity by controlling cell positioning during the developmental morphogenesis processes. Intact osteoblasts controlled the cell division axis in response to the substrate collagen orientation, leading to the alignment of daughter cells along the collagen substrates. This is consistent with the results of previous studies regarding the relationship between the cell division axis and the extracellular matrix [25]. In contrast, MDA-MB-231 cells migrated towards the osteoblasts and prevented the proper orientation of their division (Figure 4B). This is likely triggered by the impaired positioning of the centrosome caused by the direct attack of tumor cells [26]. The other possible mechanism is that the cell membrane deformation originated from the osteoblast-tumor cell attachment sites. This could be mediated by the cadherin-related intercellular dynamics, and indeed, our recent research has indicated the involvement of cadherin molecules in cancer cell-osteoblast interaction on oriented collagen substrates [17].

## 4. Materials and Methods

### 4.1. Culture of Cancer Cells

MDA-MB-231 cells (ATCC, Manassas, VA, USA) were maintained in D-MEM (GIBCO, Dublin, Ireland) containing 10% FBS, 100 U/mL penicillin, and 100 µg/mL streptomycin. MDA-PCa-2b cells (ATCC) were maintained in Ham’s F-12K (Wako, Osaka, Japan) medium containing 20% FBS, 25 ng/mL cholera toxin (Sigma, St. Louis, MO, USA), 10 ng/mL mouse epidermal growth factor (Sigma, St. Louis, MO, USA), 0.005 mM phosphoethanolamine (Sigma, St. Louis, MO, USA), 100 pg/mL hydrocortisone (Sigma), 45 nM selenious acid (Sigma, St. Louis, MO, USA), and 0.005 mg/mL bovine insulin (Sigma, St. Louis, MO, USA). All cell culture experiments were performed in accordance with the protocols provided in the cell line data sheets.

### 4.2. Ex Vivo Bone Metastasis Model

Femurs were obtained from embryonic 16.5-day-old mice (ICR; Japan SLC, Shizuoka, Japan). For the longitudinal organ culture under static loading stress, a novel culturing device was constructed through the combination of cell culture microplates (IWAKI, Shizuoka, Japan) and commercially pure (CP) titanium bases. Each well of microplate (96 well) was isolated for cultivation of individual bone tissue. To perform the uniaxial static loading with a physiological level of force, bone tissue was placed between CP titanium bases weighing 1.0 g, which possess the hollows for holding the bones between them inside the isolated well. The cancer cells (MDA-MB-231 cells and MDA-PCa-2b cells) were circulated around the bones using a magnetic stirrer at approximately 350 rpm. The circulating cancer cells were allowed to invade the bone tissue through the holes placed on the surface of the individual culture well. All animal experiments were approved by the Osaka University Committee for Animal Experimentation (approval number: 27-2-1, 25 June 2015). All experiments were performed in accordance with the related guidelines and regulations for scientific and ethical animal experimentation.

### 4.3. Bone Morphology, BMD Analysis

Femurs were scanned using micro-CT (Shimadzu, Kyoto, Japan) at X-ray energy settings of 25 kV and 130 µA, with a nominal resolution of 10 µm. The BMD (bone mineral density) was quantitatively analyzed based on the micro-CT images. The calibration curve of CT value-BMD was prepared using phantoms, which contains hydroxyapatite in the range of 200–800 kg/m^3^. The CT value was subsequently converted to BMD values using TRI-3D BON software (RATOC System Engineering, Tokyo, Japan).

### 4.4. Analysis of Apatite Orientation

The degree of apatite orientation was analyzed using a microbeam X-ray diffractometer (µXRD) system (R-Axis BQ, Rigaku, Tokyo, Japan) equipped with a transmission optical system (Mo-Kα radiation). The incident beam was radiated vertically to the long axis of the bone at a tube voltage of 50 kV and a tube current of 90 mA. The degree of preferential orientation of the c-axis in the apatite crystals was determined as the relative intensity ratio of the (002) diffraction peak to the (310) peak in the X-ray profile. This was previously reported as a suitable index for evaluating apatite orientation [4,5].

### 4.5. Immunohistochemistry

The cultured femurs were fixed in neutral buffered formaldehyde for 24 h, followed by decalcification with a 0.5 M EDTA-2Na solution (pH 7.4) for 7 days. Specimens were dehydrated through a graded series of ethanol, embedded into paraffin, and then transversely cut into 5-µm-thick sections. Deparaffinized sections were blocked with normal goat serum (ThermoFisher Scientific, Waltham, MA, USA) to block the non-specific antibody binding sites. The specimens were then incubated with mouse anti cathepsin D (Abcam, Cambridge, UK), rabbit anti MMP10 (Abcam, Cambridge, UK), mouse anti ALP (Novus Biologicals, Littleton, CO, USA), and rabbit anti osterix (Abcam, Cambridge, UK) antibodies. The secondary antibodies were as follows: Alexa Fluor 546-conjugated anti rabbit IgG (Molecular Probes, ThermoFisher Scientific, Waltham, MA, USA), Alexa Fluor 488-conjugated anti mouse IgG (Molecular Probes, ThermoFisher Scientific, Waltham, MA, USA). Nuclei were stained with DAPI (ThermoFisher Scientific, Waltham, MA, USA).

### 4.6. Osteoblast Isolation and Culture

Primary osteoblasts were isolated from the calvariae of neonatal mice. The calvariae from postnatal 2–3-day-old C57BL/6 mice were excised under aseptic conditions and placed in ice-cold α-modified Eagle’s Medium (α-MEM; GIBCO). The calvariae were then subjected to a series of collagenase/trypsin (collagenase: Wako, Osaka, Japan; trypsin: Nacalai Tesque) digestions at 37 °C for 15 min each. The supernatants obtained after the 3rd, 4th, and 5th digestion processes were neutralized with α-MEM, pooled, and filtered using a 100 µm mesh (BD Biosciences, San Jose, CA, USA). The filtrates were then centrifuged, and the resulting pellets were resuspended in α-MEM containing 10% fetal bovine serum (FBS) for cell culture. The cells were then diluted and seeded onto the fabricated specimens. 

### 4.7. Time-Lapse Imaging of the Cancer Cells and Osteoblasts Cocultured on the Oriented Collagen Substrate

Oriented collagen substrates were fabricated as described previously [6]. Porcine skin collagen type-I solution (10 mg/mL) was extruded using a three-axis robotic arm. Primary osteoblasts and cancer cells were seeded at a total cell density of 5000 cells/cm^2^. Plasmids containing pEGFP-Actin fusion constructs (Clontech, Palo Alto, CA, USA) were transfected into osteoblasts using the electroporation method (Neon, Thermofisher Scientific, Waltham, MA, USA). Cancer cells were labelled with CellTracker Red CMTPX Dye (Thermofisher Scientific, Waltham, MA, USA), according to the manufacturer’s instructions. For time-lapse recordings, the cells were kept in α-MEM containing ProLong Live Antifade Reagent (Thermofisher Scientific, Waltham, MA, USA) constantly at 37 °C and 5% CO_2_ conditions. 

### 4.8. Statistical Analysis

Statistical significance was assessed by one-way ANOVA, followed by Tukey’s post hoc test. A significance of *p* < 0.05 was required for rejection of the null hypothesis.

## 5. Conclusions

The impaired alignment of collagen/apatite in metastasized bone is the most important key factor for its mechanical dysfunction. Here, by developing a novel ex vivo metastasis model, we have shown that abnormal osteoblast arrangement is one of the principal contributors to the cancer-derived collagen/apatite disarrangement. Additionally, live imaging of osteoblasts cocultured with breast cancer cells on an oriented collagen substrate showed that the dynamic collision behavior between the tumor cells and osteoblasts plays a significant role in regulating cell alignment. Overall, our findings have highlighted that the selective regulation of the osteoblast alignment induced by cancer metastasis governs the disorganized, collapsed function of bone.
